# Mass screening and treatment (MSaT) for identifying and treating asymptomatic cases of malaria-malaria elimination demonstration project (MEDP), Mandla, Madhya Pradesh

**DOI:** 10.1186/s12936-022-04423-z

**Published:** 2022-12-27

**Authors:** Akansha Singh, Harsh Rajvanshi, Mrigendra P. Singh, Sneha Bhandari, Sekh Nisar, Rajan Poriya, Vinay Telasey, Himanshu Jayswar, Ashok K. Mishra, Aparup Das, Harpreet Kaur, Altaf A. Lal, Praveen K. Bharti

**Affiliations:** 1 Indian Council of Medical Research - National Institute of Research in Tribal Health (ICMR-NIRTH), Jabalpur, Madhya Pradesh India; 2 Malaria Elimination Demonstration Project, Mandla, Madhya Pradesh India; 3 Indian Council of Medical Research - National Institute of Research in Environment Health (ICMR-NIREH), Bhopal, Madhya Pradesh India; 4 Directorate of Health Services, Government of Madhya Pradesh, Bhopal, India; 5 Department of Health Research, Ministry of Health and Family Welfare, Indian Council of Medical Research, New Delhi, India; 6 Foundation for Disease Elimination and Control of India, Mumbai, 482003 Maharashtra India; 7 Indian Council of Medical Research - National Institute of Malaria Research (ICMR-NIMR), New Delhi, India; 8 Present Address: Asia Pacific Leaders Malaria Alliance (APLMA), Singapore, Singapore; 9 Present Address: Department of Health and Family Welfare, NHM Raigarh, Raigarh, Chattisgarh India

**Keywords:** Malaria, Mass screening and treatment, Asymptomatic malaria, Low-density infection, *Plasmodium falciparum*, *Plasmodium vivax*

## Abstract

**Background:**

Mass screening and treatment (MSaT) aims at reducing the spread of malaria in communities by identifying and treating infected persons regardless of the symptoms. This study was conducted to identify and treat asymptomatic cases using MSaT approaches in the community.

**Methods:**

Three rounds of MSaT using cluster combination approaches were carried out during September 2018 to December 2019 to identify and treat asymptomatic malaria cases in the community. All individuals who were present in the household were screened using RDT irrespective of malaria related symptoms. Simultaneously thick and thin blood smear and blood spot were collected for further analysis using microscopy and diagnostic PCR done in a subset of the samples.

**Results:**

Logistic regression analysis revealed that asymptomatic malaria cases significantly less among the older age groups compared with < 5 years children (OR ranged between 0.52 and 0.61; p < 0.05), lowest in cluster 4 (OR = 0.01; p < 0.0001); during third round of MSaT survey (OR = 0.11; p < 0.0001) and significantly higher in moderate to high endemic areas (OR = 88.30; p < 0.0001).

**Conclusion:**

Over the three rounds of MSaT, the number of asymptomatic cases were significantly less in the older age groups, and during third round. Similarly, the asymptomatic cases were significantly less in the low endemic area with API < 1 (cluster four). Therefore, the malaria elimination programme may consider the MSaT strategy to identify asymptomatic cases that would be otherwise missed by routine fever based surveillance. This MSaT strategy would help accomplish the malaria elimination goal in an expedited manner.

## Background

India has committed to the global malaria elimination goal of 2030, which translates to achieving zero indigenous cases by 2027 [1]. Over the last two decades, malaria incidence has significantly reduced in the South East Asia Region from 23 million cases in 2000 to ~ 5 million cases in 2020. However, India still accounted for 83% of malaria cases and 82% of malaria fatalities in the region [2]. Some of the challenges towards malaria elimination in India include diverse climate, diverse vector population, complex epidemiology, emerging drug and insecticide resistance, asymptomatic malaria, low-density infections and various operational limitations [3–8].

The Mass Screening and Treatment (MSaT) aims to detect and treat all malarial parasite infections, including asymptomatic parasite carriers within the community. The World Health Organization recommends MSaT as an intervention strategy targeting asymptomatic infections to reduce the prevalence [9]. In another study that summarizes the outcomes of advisory board discussions concluded that treatment of asymptomatic carriers with ACT may help resolve the infection reservoirs [10]. The success of MSaT would depend on the population coverage and use of highly-sensitive diagnostic tools [11, 12]. Rapid diagnostic tests (RDTs) have been the ‘tool of choice’ for MSaT and have reported sensitivity and specificity similar to microscopy performed by a competent microscopist [11]. Compared to RDT and microscopy, the Polymerase Chain Reaction (PCR) technique is more sensitive and has been widely used for diagnosis, epidemiological, and drug efficacy assessments [13–15].

In the present study, three rounds of MSaT were conducted using RDTs, microscopy, and PCR to identify asymptomatic malaria-infected individuals. The MSaT was accompanied by vector control interventions such as LLIN and IRS, along with active fever surveillance and case management. These three round of MSaT were done during the pre-transmission, transmission, and post-transmission period for identifying and treating the asymptomatic cases in the community of district Mandla, Madhya Pradesh, India.

## Methods

This study was part of the Malaria Elimination Demonstration Project, which was a first-of-its-kind public–private-partnership between the Indian Council of Medical Research (ICMR) through the National Institute for Research in Tribal Health (NIRTH) Jabalpur, Government of Madhya Pradesh (GoMP), and the Foundation for Disease Elimination and Control of India (established by Sun Pharmaceutical Industries Ltd. as a not-for-profit entity).

### Study site

The study was conducted in district Mandla, located between coordinates of 22°02′ and 23°22′ N latitudes and 80°18′ and 81°50′ E longitudes in the east-central region of the state of Madhya Pradesh (MP), India. The total geographical area of the district was 8771 km^2^, inhabited by ethnic tribal communities, mainly *‘Gonds’* and *‘Baiga’*.

### Study design and sampling method

A cross sectional MSaT was conducted during the malaria transmission season in September 2018, pre-transmission season in June 2019 and post-transmission season in December 2019 to assess the asymptomatic cases of malaria in the community. A multistage sampling method was adapted. In September 2018, for the first round of MSaT, a cluster sampling method was used in which all the villages of the district Mandla were divided into four clusters based on their Annual Parasite Incidence (API: number of malaria positive cases per thousand population in one calendar year) during five preceding years (2013–2017) and accessibility to the study areas.

The clusters were categorized as: (1) Hard-to-reach or inaccessible villages; (2) More than or equal to API 5; (3) API between 1 to 4.99; and (4) API between 0 and 0.99. The second round of the MSaT survey was conducted in June 2019 to validate the findings from the first round of MSaT and the entire study area was further stratified into moderate to high endemic (> 1 API) and low endemic (0–1 API) villages. Further, a third round of the MSaT survey was carried out in December 2019 in 50 houses surrounding the cryptic cases [16] diagnosed in these villages.

### Sample size

Sample size was determined using the formula of simple random sampling for a finite population as given below:

$$n=\frac{\frac{{z}^{2}p\left(1-p\right)}{{e}^{2}}}{1+(\frac{{z}^{2}p\left(1-p\right)}{{e}^{2}N})}$$

where, n is the required sample size; z = 1.96 at 95% confidence interval; p is the considered probability of asymptomatic malaria prevalence; e is the marginal precision; and N is the population size.

The sample size for the different clusters and malaria endemic areas was determined based on the lowest asymptomatic malaria prevalence reported by Wangchuk et al. [17] in Bhutan [0.27% (95% CI: 0.05–1.07)]. The 50% relative precision was considered, and the population of the district Mandla was taken at 1100000. Further, this sample was multiplied by 1.5 as a design effect and inflated to a 30% non-response. The sample guided screening of individuals for malaria, irrespective of any malaria-related symptoms in 3700; 9000; 9500 and 12500 individuals in clusters 1 to 4, respectively; and 6600 and 12000 in moderate to high and low endemic areas, respectively.

### Mass screening and treatment (MSaT)

Door-to-door visits were done by trained Village Malaria Workers (VMWs) to screen the community for malaria parasites. The VMWs screened each consenting individual in the study area using bivalent malaria RDTs (SD Bioline Malaria Ag Pf/Pv. All positive cases were treated with anti-malarials as per the national treatment guidelines [18].

Alongside RDT screening, both thick and thin blood smears were collected and stained using JSB solution for microscopic examination and three drop of blood was spotted on Whatman no. 3 paper for molecular analysis. When no parasites were observed after examination of 100 thick fields containing at least 10 white blood cells (WBCs) per field, a blood slide was considered negative. All the samples having discordant results were re-checked by an independent WHO certified microscopist for quality assurance of light microscopy.

### Molecular diagnosis

Molecular analysis was done in about one-third of the randomly selected samples. Genomic DNA was isolated from the dried blood spots using the Chelex method [Chelex-100 Sodium form (50–100 Mesh) Himedia Laboratories [19]. The presence of *Plasmodium* species was determined using species specific nested PCR by targeting 18Sr RNA gene. To set up the primary PCR, 5 µL of genomic DNA as template was taken to amplify the 18S rRNA gene for the *Plasmodium* genus using forward and reverse primer [15]. The primary PCR product was diluted 1:10 times and used for the nested PCR which was performed using four different species-specific primer pairs for *Plasmodium falciparum, Plasmodium vivax, Plasmodium malariae* and *Plasmodium ovale.* The PCR product was analysed on 1.2% Agarose gel electrophoresis.

### Statistical analysis

The demographic (age, gender, and area of residence) variables, clinical symptoms related to malaria, results of RDT, microscopy, and PCR were entered in Microsoft Excel 2007 worksheet, and the numerically coded data was exported in R 4.1.2 for Windows (R Foundation for Statistical Computing, Vienna, Austria.) for statistical analysis. Logistic regression analysis was used to examine the association of independent variable(s).

## Results

A total of 88142 individuals, irrespective of malaria-related symptoms, were tested using RDTs during door-to-door visits. Out of which, 34694 (39.36%), 18550 (21.05%) and 34898 (39.59%) were screened in the first, second and third rounds of the MSaT surveys, respectively. In clusters one to four; 4437 (5.03%), 17616 (19.99%), 9709 (11.02%) and 56380 (63.96%) individuals were screened, respectively. About three-fourths of the individuals were adults with a mean age of 31.86 ± 20.07 years, and about 51% were female (Table 1).

**Table 1:** Demographic characteristics of the individuals tested during mass survey and treatment

	Cluster 1 (Hard to reach)	Cluster 2 (API: ≥ 5)	Cluster 3 (API: 1–4.99)	Cluster 4 (API: 0–1)	Total
Age					
0–5 yrs	372 (8.38)	1403 (7.96)	759 (7.82)	3766 (6.68)	6300 (7.15)
5–15 yrs	807 (18.19)	3377 (19.17)	1914 (19.71)	10,563 (18.74)	16,661 (18.90)
15–25 yrs	710 (16.00)	3118 (17.70)	1811 (18.65)	9570 (16.97)	15,209 (17.26)
25–50 yrs	1663 (37.48)	6533 (37.09)	3747 (38.59)	19,822 (35.16)	31,765 (36.04)
Above 50 yrs	885 (19.95)	3185 (18.08)	1478 (15.22)	12,659 (22.45)	18,207 (20.66)
Total	4437	17,616	9709	56,380	88,142
Sex					
Female	2318 (52.24)	8906 (50.56)	4901 (50.48)	28,649 (50.81)	44,774 (50.80)
Male	2119 (47.76)	8710 (49.44)	4808 (49.52)	27,731 (49.19)	43,368 (49.20)
Total	4437	17,616	9709	56,380	88,142

During the three rounds of MSaT, the overall prevalence of malaria diagnosed by RDT or microscopy was 0.15% (132/88142). Out of which, 0.20% (69/34694), 0.15% (28/18550), and 0.10% (35/34898) cases were recorded during the first, second and third rounds of MSaT, respectively, with a statistically significant declining trend (p = 0.003).

The cluster-wise (one to four) prevalence of malaria was 1.19% (53/4437), 0.28% (50/17616), 0.29% (28/9709), and 0.002% (1/56380), respectively, which was statistically significant (p < 0.0001). Amongst the residents of low malaria endemic areas, the prevalence was 0.002% (1/56380). Whereas the prevalence in moderate-to-high malaria endemic areas was 0.41% (131/31762), which was significantly higher (p < 0.0001). The PCR based diagnosis was done in a sample of 24357, which was randomly selected from the total 88142 MSaT samples. Out of these 24357 cases tested by PCR, 23681 were asymptomatic, and the rest 676 were symptomatic. Amongst the samples tested by PCR, the malaria positive cases by RDT were 0.12% (30/24357), microscopy were 0.14% (35/ 24357), and PCR were 1.06% (258/24357).

The prevalence of asymptomatic malaria infections in the first, second, and third rounds was 0.53% (179/34013), 0.57% (103/18202) and 0.06% (20/33797), respectively. Further, analysis revealed that the asymptomatic malaria prevalence significantly declined during the third round as compared to the first round (OR = 0.11; 95% CI: 0.07–0.18; p < 0.0001). Similar declining trend was recorded in all the clusters (p < 0.0001). Neither asymptomatic nor symptomatic malaria cases was reported from clusters three (low endemic) and four (moderate endemic) during the second and third rounds of the MSaT survey (Table 2).

**Table 2:** Prevalence of asymptomatic and symptomatic malaria in four clusters of district Mandla during three rounds of mass screening and treatment surveys

Round	Asymptomatic					Symptomatic					Total				
	Cluster 1	Cluster 2	Cluster 3	Cluster 4	Total	Cluster 1	Cluster 2	Cluster 3	Cluster 4	Total	Cluster 1	Cluster 2	Cluster 3	Cluster 4	**Total**
	(Hard-to-Reach)	(API: ≥ 5)	(API: 1–4.99)	(API: 0–1)		(Hard-to-reach)	(API: ≥ 5)	(API: 1–4.99)	(API: 0–1)		Hard-to-Reach)	(API: ≥ 5)	(API: 1–4.99)	(API: 0–1)	
	n/d (%)	n/d (%)	n/d (%)	n/d (%)	n/d (%)	n/d (%)	n/d (%)	n/d (%)	n/d (%)	n/d (%)	n/d (%)	n/d (%)	n/d (%)	n/d (%)	n/d (%)
1	9/3648 (0.25)	90/8772 (1.03)	74/9119 (0.81)	6/12474 (0.05)	179/34013 (0.53)	2/67 (2.98)	9/199 (4.52)	7/302 (2.32)	0/113 (0.00)	18/681 (2.64)	11/3715 (0.30)	99/8971 (1.10)	81/9421 (0.86)	6/12587 (0.05)	197/34694 (0.57)
2	16/135 (11.85)	87/6184 (1.41)	0/185 (0.00)	0/11698 (0.00)	103/18202 (0.57)	0/0	0/127 (0.00)	0/6 (0.00)	0/215 (0.00)	0/348 (0.00)^a^	16/135 (11.85)	87/6311 (1.38)	0/191 (0.00)	0/11913 (0.00)	103/18550 (0.55)
3	18/565 (3.19)	2/2313 (0.09)	0/96 (0.00)	0/30823 (0.00)	20/33797 (0.06)^b^	17/22 (77.27)	17/21 (80.95)	0/1 (0.00)	0/1057 (0.00)	34/1101 (3.09)	35/587 (5.96)	19/2334 (0.81)	0/97 (0.00)	0/31880 (0.00)	54/34898 (0.15)
Total	43/4348 (0.99)	179/17269 (1.04)	74/9400 (0.79)	6/54995 (0.01)	302/86012 (0.35)	19/89 (21.35)	26/347 (7.49)	7/309 (2.26)	0/1385 (0.00)	52/2130 (2.44)	62/4437 (1.40)	205/17616 (1.16)	81/9707 (0.83)	6/56380 (0.01)	354/88142 (0.40)

n/d: numerator (malaria positive)/denominator (number tested). (The malaria positive cases were diagnosed by either/or any diagnostic methods which included on spot diagnosis by RDT, light microscopy of blood smears and PCR)

The prevalence of symptomatic malaria decreased significantly during the second round 0% (0/348) than first round 2.64% (18/681) of MSaT survey (p = 0.002), while it increased in the third round (3.09%, 34/1101) but not statistically significant. A similar trend was reported in clusters one and two (hard to reach and high endemic areas). None of the symptomatic malaria cases was reported in cluster three and four during second and third round of MSaT survey (Table 2).

Malaria prevalence among asymptomatic cases diagnosed by microscopy, RDT and PCR was 0.32% (14/4348), 0.48% (21/4348) and 0.89% (12/1347) in cluster one; 0.14% (24/1726), 0.10% (18/1726) and 1.33% (151/11348) in cluster two; 0.16% (15/9400), 0.08% (8/9400) and 2.72% (64/2349) in cluster three; and 0% (0/54995), 0.002% (1/54995) and 0.06% (5/8637) in cluster four, respectively. The analysis revealed that a significantly higher prevalence of asymptomatic malaria was reported using PCR diagnosis in all the clusters (p < 0.0001).

This prevalence varied significantly over the MSaT rounds through all the clusters and diagnostic methods. Among all the diagnostic methods, the asymptomatic malaria prevalence significantly declined in the third MSaT round as compared to the first MSaT round (p < 0.001) except in cluster one, where the asymptomatic malaria prevalence was found to be significantly higher in the third round than the first round (p < 0.001). However, a higher prevalence of symptomatic malaria was found during the third round of MSaT as compared to the first round in all the diagnosis methods, and it was significant in cases diagnosed using RDT and PCR (p < 0.001) (Table 3).

**Table 3:** Cluster wise Prevalence of asymptomatic and symptomatic malaria diagnosed by RDT, microscopy and PCR in district Mandla during three rounds of mass screening and treatment surveys

Diagnostic method	Round	Asymptomatic [n/d (%)]					Symptomatic [n/d (%)]					Total				
		Cluster 1	Cluster 2	Cluster 3	Cluster 4	Total	Cluster 1	Cluster 2	Cluster 3	Cluster 4	Total	Cluster 1	Cluster 2	Cluster 3	Cluster 4	Total
		Hard to reach	API ≥ 5	API 1–4.99	API 0–1		Hard to reach	API ≥ 5	API 1–4.99	API 0–1		Hard to reach	API ≥ 5	API 1–4.99	API 0–1	
Microscopy	1	4/3648 (0.11)	11/8772 (0.12)	15/9119 (0.16)	0/12474 (0.00)	30/34013 (0.09)	0/67 (0.00)	0/199 (0.00)	1/302 (0.33)	0/113 (0.00)	1/681 (0.15)	4/3715 (0.11)	11/8971 (0.12)	16/9421 (0.17)	0/12587 (0.00)	31/34694 (0.09)
	2	4/135 (2.96)****	12/6184 (0.19)	0/185 (0.00)	0/11698 (0.00)	16/18202 (0.09)	0/0 (0.00)	0/127 (0.00)	0/6 (0.00)	0/215 (0.00)	0/348 (0.00)	4/135 (2.96)	12/6311 (0.19)	0/191 (0.00)	0/11913 (0.00)	16/18550 (0.09)
	3	6/565 (1.06)****	1/2313 (0.04)	0/96 (0.00)	0/30823 (0.00)	7/33797 (0.02)***	10/22 (45.45)	1/21 (4.76)	0/1 (0.00)	0/1057 (0.00)	11/1101 (1.00)	16/587 (2.73)	2/2334 (0.09)	0/97 (0.00)	0/31880 (0.00)	18/34898 (0.05)
	Total	14/4348 (0.32)	24/17269 (0.14)	15/9400 (0.16)	0/54995 (0.00)	53/86012 (0.06)	10/89 (11.24)	1/347 (0.29)	1/309 (0.32)	0/1385 (0.00)	12/2130 (0.56)	24/4437 (0.54)	25/17616 (0.14)	16/9709 (0.16)	0/56380 (0.00)	65/88142 (0.07)
RDT	1	3/3648 (0.08)	13/8772 (0.15)	8/9119 (0.09)	1/12474 (0.01)	25/34013 (0.07)	2/67 (2.98)	7/199 (3.52)	5/302 (1.66)	0/113 (0.00)	14/681 (2.06)	5/3715 (0.13)	20/8971 (0.22)	13/9421 (0.14)	1/12587 (0.01)	39/34694 (0.11)
	2	7/135 (5.18)****	5/6184 (0.08)	0/185 (0.00)	0/11698 (0.00)	12/18202 (0.07)	0/0 (0.00)	0/127 (0.00)	0/6 (0.00)	0/215 (0.00)	0/348 (0.00)	7/135 (5.19)	5/6311 (0.08)	0/191 (0.00)	0/11913 (0.00)	12/18550 (0.06)
	3	11/565 (1.95)****	0/2313 (0.00)	0/96 (0.00)	0/30823 (0.00)	11/33797 (0.03)*	6/22 (27.27)**	0/21 (4.76)	0/1 (0.00)	0/1057 (0.00)	6/1101 (0.54)**	17/587 (2.90)	0/2334 (0.00)	0/97 (0.00)	0/31880 (0.00)	17/34898 (0.05)
	Total	21/4348 (0.48)	18/17269 (0.10)	8/9400 (0.08)	1/54995 (0.002)	48/86012 (0.06)	8/89 (8.99)	7/347 (2.02)	5/309 (1.62)	0/1385 (0.00)	20/2130 (0.94)	29/4437 (0.65)	25/17616 (0.14)	13/9709 (0.13)	1/56380 (0.00)	68/88142 (0.08)
PCR	1	5/1277 (0.39)	72/5392 (1.33)	64/2311 (2.77)	5/2556 (0.20)	146/11536 (1.27)	0/37 (0.00)	3/134 (2.24)	2/102 (1.96)	0/33 (0.00)	5/306 (1.63)	5/1314 (0.38)	75/5526 (1.36)	66/2413 (2.74)	5/2589 (0.19)	151/11842 (1.28)
	2	6/53 (11.32)****	77/4641 (1.66)	0/18 (0.00)	0/68 (0.00)	83/4780 (1.74)*	0/0 (0.00)	0/117 (0.00)	0/0 (0.00)	0/0 (0.00)	0/117 (0.00)	6/53 (11.32)	77/4758 (1.62)	0/18 (0.00)	0/68 (0.00)	83/4897 (1.69)
	3	1/17 (5.88)*	2/1315 (0.15)**	0/20 (0.00)	0/6013 (0.00)	3/7365 (0.04)****	4/4 (100.0)	17/17 (100.0)	0/0 (0.00)	0/232 (0.00)	21/253 (8.30)***	5/21 (23.81)	19/1332 (1.43)	0/20 (0.00)	0/6245 (0.00)	24/7618 (0.32)
	Total	12/1347 (0.89)	151/11348 (1.33)	64/2349 (2.72)	5/8637 (0.06)	232/23681 (0.98)	4/41 (9.76)	20/268 (7.46)	2/102 (1.96)	0/265 (0.00)	26/676 (3.85)	16/1388 (1.15)	171/11616 (1.47)	66/2451 (2.69)	5/8902 (0.06)	258/24357 (1.06)

n/d: numerator (malaria positive)/denominator (number tested)

^*^p < 0.05

^**^p < 0.01

^***^p < 0.001

^****^p < 0.0001

Univariate logistic regression analysis revealed that asymptomatic malaria was significantly lower among the age groups 5–15 years (OR = 0.60; 95% CI = 0.39–0.92; p < 0.05), 15–25 years (OR = 0.52; 95% CI = 0.33–0.81; p < 0.01), 25–50 years (OR = 0.61; 95% CI = 0.41–0.89; p < 0.05) and above 50 years (OR = 0.54; 95% CI = 0.36–0.83; p < 0.01) in reference to the < 5 years children. Further analysis showed asymptomatic malaria was significantly lower in cluster four (OR = 0.01; 95% CI = 0.005–0.026; p < 0.0001), during the third round of MSaT survey (OR = 0.11; 95% CI = 0.07–0.18; p < 0.0001) and significantly higher in the moderate to high endemic areas (OR = 88.30; 95% CI = 39.35–198.17; p < 0.0001) than their reference categories. However, the asymptomatic malaria cases did not significantly differ between age groups and gender (p > 0.05). Further, symptomatic malaria cases were significantly lower in the clusters two (OR = 0.30; 95% CI = 0.16–0.57; p < 0.0001) and three (OR = 0.08; 95% CI = 0.03–0.21; p < 0.0001) as compared to the cluster one.

Symptomatic malaria cases did not differ significantly through different age groups, gender, endemicity and rounds of the MSaT surveys (p > 0.05). The total malaria prevalence in MSaT significantly decreased among the older age groups than < 5 years children (p < 0.05); in round three (OR = 0.27; 95% CI = 0.20–0.37; p < 0.0001); in cluster two (OR = 0.59; 95% CI = 0.43–0.83; p < 0.001) and cluster four (OR = 0.01; 95% CI = 0.003–0.02; p < 0.0001) than their reference categories, while it was 104 times likely to be higher in moderate-to-high endemic areas than low endemic areas (Table 4).

**Table 4:** Logistic regression analysis of factors associated with asymptomatic and symptomatic malaria in MSaT survey

Factors	Asymptomatic	Symptomatic	Total
	OR (95% CI)	OR (95% CI)	OR (95% CI)
Age groups (years)			
< 5	Reference	Reference	Reference
5–15	0.60 (0.39–0.92)*	2.58 (0.69–9.66)	0.66 (0.44–0.98)*
15–25	0.52 (0.33–0.81)**	2.88 (0.78–10.59)	0.60 (0.39–0.90)*
25–50	0.61 (0.41–0.89)*	2.24 (0.67–7.52)	0.69 (0.48–0.99)*
Above 50	0.54 (0.36–0.83)**	1.29 (0.33–5.03)	0.57 (0.38–0.86)**
Gender			
Female	Reference	Reference	Reference
Male	0.94 (0.75–1.18)	1.48 (0.85–2.57)	0.99 (0.80–1.22)
Clusters			
Cluster 1 (Hard to reach)	Reference	Reference	Reference
Cluster 2 (API ≥ 5)	1.05 (0.75–1.46)	0.30 (0.16–0.57)****	0.59 (0.43–0.83)**
Cluster 3 (API 1–4.99)	0.79 (0.54–1.16)	0.08 (0.03–0.21)****	0.83 (0.62–1.11)
Cluster 4 (API 0–1)	0.01 (0.005–0.026)****	empty	0.01 (0.003–0.02)****
Round			
1	Reference	Reference	Reference
2	1.07 (0.84–1.37)	empty	0.98 (0.77–1.24)
3	0.11 (0.07–0.18)****	1.17 (0.66–2.09)	0.27 (0.20–0.37)****
Endemicity			
Low	Reference	Reference	Reference
Moderate to high	88.30 (39.35–198.17)****	omitted	104.08 (46.43–233.30)****

*OR*: odds ratio, *CI*: confidence intervals

^*^p < 0.05

^**^p < 0.01

^***^p < 0.001

^****^p < 0.0001

## Discussion

India is progressing towards the goal of malaria elimination by 2030, in view of complex and varied vector bionomics, supply chain, inaccessible populations, natural calamities, and climate change [20, 21]. Besides the existing package of malaria interventions, such as the long-lasting insecticidal nets (LLINs), indoor residual spraying (IRS), and early diagnosis and treatment, the MSaT provides an additional tool that has the potential to reduce malaria burden by detecting cases that would be missed by routine fever-based surveillance.

The state of Odisha reported a ~ 95% decline in malaria cases from 2016 to 2021 using MSaT and LLINs, particularly in the inaccessible tribal areas [21]. The state of Tripura also significantly reduced its malaria burden in recent years through MSaT and LLINs [8]. The state of Chhattisgarh initiated the *Malaria Mukt Bastar* campaign, which used MSaT as one of the key interventions in the tribal areas of Bastar to reduce malaria morbidity [22].

The present study was conducted as part of the Malaria Elimination Demonstration Project and used a cluster combination approaches for three rounds of MSaT from September 2018, June 2019 to December 2019. The malaria prevalence diagnosed either by RDT or microscopy showed a significant declining trend, 0.20% (first round), 0.15% (second round), and 0.10% (third round). This observation was similar to a study done in Zambia that revealed 17% reduction in the incidence of clinical malaria after three rounds of MSaT [12]. The group pointed out that the conventional RDTs would prevent the detection of a significant reservoir of low-density infections that might contribute to maintaining transmission in the community. The updated recommendations from WHO in November 2022 have informed that Mass Testing and Treatment (MTaT) may be done using RDTs, microscopy, and nucleic-acid-based tests [23]. In this context, recently published the results of a study designed to estimate prevalence of low-density sub-RDT and sub-microscopy infections, which revealed 1.4% higher infections diagnosed using PCR compared to RDTs and 1.8% as compared to microscopy [24]. The results of the present study reveal that a large number of asymptomatic cases are missed by RDTs, which suggests that a diagnostic test, such as nucleic acid tests with higher specificity and sensitivity, is needed to identify the true burden of malaria cases at the community level.

The present study revealed a significantly higher prevalence of asymptomatic infections in moderate to high endemic areas (0.41%) as compared to the low endemic areas (0.0%). Compared to this, an opposite result was found in western Kenya, but the sample size was much lower [25].

The hard-to-reach areas remain a bottleneck of malaria elimination due to inadequate health care services/ service providers. The cluster wise analysis of the present study reported a prevalence of 1.19% (hard to reach area), 0.28% (API: > 5), 0.29% (API: 1–4.99), and 0.002% (API: 0–1) with a declining trend over different rounds (p < 0.0001). A study known as the ‘*Durgama Anchalare Malaria Nirakarana* (DAMaN)’ focussing on these hard-to-reach areas was conducted in Odisha and reported a significant reduction in malaria cases using MSaT, LLINs, and community mobilisation [26].

In another MSaT study conducted in Malawi in school children, a decrease in malaria transmission was reported [27], which is similar to the present study. Another study conducted in Northern Senegal reported a 38% decrease in malaria case incidence [28].

The present study has revealed that the asymptomatic malaria infections were significantly reduced in the third round 0.06% of MSaT as compared to the first round (0.53%), which suggest that MSaT should be considered as a strategy along with other case management and vector control tools for effectively halting malaria transmission in the community.

Another important observation of this study is the finding that PCR detects more than three times higher cases as compared to RDT / microscopy. The asymptomatic cases diagnosed by PCR were higher in all clusters than the conventional methods and were highest in moderate to high endemic areas. In a similar study from Zanzibar, it was found that MSaT detected more than 10 times higher cases using species-specific PCR (2.5%) as compared to RDT (0.2%) [11]. This finding raises concern over the limitations of RDTs and their usage as the only diagnostic tool for malaria elimination campaigns [29].

The usefulness of a sensitive point-of-care PCR-based diagnostic tool for MSaT was highlighted by Von Seidlein L [30], which is in agreement with the findings of this study. Similarly, Hoyer et al.tested a strategy known as ‘Focused Screening and Treatment (FSaT)’ to detect, treat and track clusters of asymptomatic carriers of *P. falciparum* using PCR tools [31], and reported a significant decrease in the prevalence of symptomatic malaria during the second round of MSaT survey.

The evidence generated by this Malaria Elimination Demonstration Project [3, 24, 32–41] indicates that surveillance, case management, and vector control need the full attention of programmes for eliminating malaria. However, in specific scenarios, such as hard-to-reach areas, pockets of high transmission, and incidence of disease outbreaks, MSaT/MTaT/FSaT might be considered on a priority basis. This additional case management tool would hit at asymptomatic infections, which routine strategies such as fever-based surveillance otherwise will miss.

## Conclusion

This study has demonstrated the significance of MSaT as an effective surveillance tool to detect and treat asymptomatic cases of malaria. Given the limitations of RDT and microscopy, it is recommended that national programmes and the WHO should implement a policy that recommends the use of nucleic acid-based detection tools, such as PCR, for detecting asymptomatic reservoirs of malaria during elimination phase.

## Data Availability

We have reported all the findings in this manuscript. The hardcopy data is stored at MEDP Office in Jabalpur, Madhya Pradesh, and Indian Council of Medical Research-National Institute of Research in Tribal Health (ICMR-NIRTH), Jabalpur, Madhya Pradesh. Softcopy data is available on the project server of MEDP hosted by Microsoft Azure. If anyone wants to review or use the data, they should contact: Dr. Altaf A. Lal Project Director—Malaria Elimination Demonstration Project, Mandla Foundation for Disease Elimination and Control of India, Mumbai, India 482003.

## References

[1] WHO (2015). Global technical strategy for malaria 2016–2030.

[2] WHO (2021). World malaria report 2021.

[3] Bharti PK, Rajvanshi H, Nisar S, Jayswar H, Saha KB, Shukla MM (2020). Demonstration of indigenous malaria elimination through track-test-treat-track (T4) strategy in a Malaria elimination demonstration project in Mandla. Madhya Pradesh Malar J.

[4] Chaturvedi N, Krishna S, Bharti PK, Gaur D, Chauhan VS, Singh N (2017). Prevalence of afebrile parasitaemia due to Plasmodium falciparum & *P. vivax* in district Balaghat (Madhya Pradesh): Implication for malaria control. Indian J Med Res.

[5] van Eijk AM, Sutton PL, Ramanathapuram L, Sullivan SA, Kanagaraj D, Priya G (2019). The burden of submicroscopic and asymptomatic malaria in India revealed from epidemiology studies at three varied transmission sites in India. Sci Rep.

[6] Kumari P, Sinha S, Gahtori R, Yadav CP, Pradhan MM, Rahi M (2020). Prevalence of asymptomatic malaria parasitemia in Odisha, India: a challenge to malaria elimination. Am J Trop Med Hyg.

[7] Kaura T, Kaur J, Sharma A, Dhiman A, Pangotra M, Upadhyay A (2019). Prevalence of submicroscopic malaria in low transmission state of Punjab: a potential threat to malaria elimination. J Vector Borne Dis.

[8] Bhowmick IP, Nirmolia T, Pandey A, Subbarao SK, Nath A, Senapati S (2021). Dry post wintertime mass surveillance unearths a huge burden of *P. vivax*, and mixed infection with *P. vivax*
*P. falciparum*, a threat to malaria elimination, in Dhalai, Tripura India. Pathogens..

[9] WHO. Global plan for artemisinin resistance containment (GPARC). Geneva, World Health Organization; 2011.

[10] Ogutu B, Tiono AB, Makanga M, Premji Z, Gbadoé AD, Ubben D (2010). Treatment of asymptomatic carriers with artemether-lumefantrine: an opportunity to reduce the burden of malaria?. Malar J.

[11] Cook J, Xu W, Msellem M, Vonk M, Bergström B, Gosling R (2015). Mass screening and treatment on the basis of results of a *Plasmodium falciparum-*specific rapid diagnostic test did not reduce malaria incidence in Zanzibar. J Infect Dis.

[12] Larsen DA, Bennett A, Silumbe K, Hamainza B, Yukich JO, Keating J (2015). Population-wide malaria testing and treatment with rapid diagnostic tests and artemether-lumefantrine in southern Zambia: a community randomized step-wedge control trial design. Am J Trop Med Hyg.

[13] Snounou G, Viriyakosol S, Zhu XP, Jarra W, Pinheiro L, Rosario VE (1993). High sensitivity of detection of human malaria parasites by the use of nested polymerase chain reaction. Mol Biochem Parasitol.

[14] Nema S, Singh A, Krishna S, Poriya R, Dubey S, Ali NA (2022). Unreported mixed *Plasmodium* species infection may increase vivax malaria in India: a challenge for malaria elimination. Trans R Soc Trop Med Hyg.

[15] Krishna S, Bharti PK, Chandel HS, Ahmad A, Kumar R, Singh PP, et al. Detection of mixed infections with *Plasmodium* spp. by PCR, India, 2014. Emerg Infect Dis. 2015;21:1853–7. 10.3201/eid2110.150678 PMC4593445 26401635

[16] Centers for disease control and prevention. Glossary: malaria. https://www.cdc.gov/malaria/glossary.html

[17] Wangchuk S, Gyeltshen S, Dorji K, Wangdi T, Dukpa T, Namgay R (2019). Malaria elimination in Bhutan: asymptomatic malaria cases in the Bhutanese population living in malaria-risk areas and in migrant workers from India. Rev Inst Med Trop Sao Paulo.

[18] National vector borne disease control programme. Ministry of health and family welfare, Government of India. National drug policy on malaria. https://www.nvbdcp.gov.in/Doc/National-Drug-Policy-2013.pdf

[19] Plowe CV, Djimde A, Bouare M, Doumbo O, Wellems TE (1995). Pyrimethamine and proguanil resistance-conferring mutations in *Plasmodium falciparum* dihydrofolate reductase: polymerase chain reaction methods for surveillance in Africa. Am J Trop Med Hyg.

[20] Rahi M, Sharma A (2022). Malaria control initiatives that have the potential to be gamechangers in India's quest for malaria elimination. Lancet Regional Health-Southeast Asia.

[21] Pradhan MM, Meherda P (2019). Malaria elimination drive in Odisha: hope for halting the transmission. J Vector Borne Dis.

[22] Ghosh SK, Ghosh C. Innovations in vector-borne disease control in India In Public health in developing countries-challenges and opportunities Anugwom EE, Awofeso N (eds) IntechOpen Publ. 2020.

[23] WHO. Guidelines for malaria v5.4. Geneva, World Health Organization; 2022. https://app.magicapp.org/#/guideline/LwRMXj/section/EPNbAq

[24] Singh A, Singh MP, Bhandari S, Rajvanshi H, Nisar S, Telasey V (2022). Significance of nested PCR testing for the detection of low-density malaria infection amongst febrile patients from the Malaria elimination demonstration project in Mandla, Madhya Pradesh. India Malar J.

[25] Desai MR, Samuels AM, Odongo W, Williamson J, Odero NA, Otieno K (2020). Impact of intermittent mass testing and treatment on incidence of malaria infection in a high transmission area of Western Kenya. Am J Trop Med Hyg.

[26] Bal M, Das A, Ghosal J, Pradhan MM, Khuntia HK, Pati S (2020). Assessment of effectiveness of DAMaN: a malaria intervention program initiated by Government of Odisha India. PLoS ONE.

[27] Cohee LM, Valim C, Coalson JE, Nyambalo A, Chilombe M, Ngwira A (2021). School-based screening and treatment may reduce *P. falciparum* transmission. Sci Rep.

[28] Conner RO, Dieye Y, Hainsworth M, Tall A, Cissé B, Faye F (2020). Mass testing and treatment for malaria followed by weekly fever screening, testing and treatment in Northern Senegal: feasibility, cost and impact. Malar J.

[29] Bharti PK, Chandel HS, Ahmad A, Krishna S, Udhayakumar V, Singh N (2016). Prevalence of pfhrp2 and/or pfhrp3 gene deletion in *Plasmodium falciparum* population in eight highly endemic states in India. PLoS ONE.

[30] von Seidlein L (2014). The failure of screening and treating as a malaria elimination strategy. PLoS Med.

[31] Hoyer S, Nguon S, Kim S, Habib N, Khim N, Sum S (2012). Focused screening and treatment (FSAT): a PCR-based strategy to detect malaria parasite carriers and contain drug resistant *P. falciparum*, Pailin Cambodia. PLoS ONE.

[32] Rajvanshi H, Bharti PK, Nisar S, Jain Y, Jayswar H, Mishra AK (2020). Study design and operational framework for a community-based malaria elimination demonstration project (MEDP) in 1233 villages of district Mandla. Madhya Pradesh Malar J.

[33] Sharma RK, Rajvanshi H, Bharti PK, Nisar S, Jayswar H, Mishra AK (2021). Socio-economic determinants of malaria in tribal dominated mandla district enrolled in Malaria Elimination Demonstration Project in Madhya Pradesh. Malar J.

[34] Rajvanshi H, Nisar S, Bharti PK, Jayswar H, Mishra AK, Sharma RK (2021). Significance of training, monitoring and assessment of malaria workers in achieving malaria elimination goal of malaria elimination demonstration project. Malar J.

[35] Mishra AK, Bharti PK, Vishwakarma A, Nisar S, Rajvanshi H, Sharma RK (2020). A study of malaria vector surveillance as part of the Malaria Elimination Demonstration Project in Mandla. Madhya Pradesh Malar J.

[36] Mishra AK, Nisar S, Rajvanshi H, Bharti PK, Saha KB, Shukla MM (2021). Improvement of indoor residual spraying and long-lasting insecticidal net services through structured monitoring and supervision as part of the malaria elimination demonstration project in Mandla. Madhya Pradesh Malar J.

[37] Rajvanshi H, Saha KB, Shukla MM, Nisar S, Jayswar H, Mishra AK (2021). Assessment of ASHA for knowledge, diagnosis and treatment on malaria in Mandla district of Madhya Pradesh as part of the malaria elimination demonstration project. Malar J.

[38] Rajvanshi H, Bharti PK, Nisar S, Jayswar H, Mishra AK, Sharma RK (2021). A model for malaria elimination based on learnings from the malaria elimination demonstration project, Mandla district. Madhya Pradesh Malar J.

[39] Rajvanshi H, Jain Y, Kaintura N, Soni C, Chandramohan R, Srinivasan R (2021). A comprehensive mobile application tool for disease surveillance, workforce management and supply chain management for malaria elimination demonstration project. Malar J.

[40] Rajvanshi H, Mishra K, Bharti PK, Sandhibigraha D, Nisar S, Jayswar H (2021). Learnings from two independent malaria elimination demonstration projects in India. Trans R Soc Trop Med Hyg.

[41] Rajvanshi H, Bharti PK, Sharma RK, Nisar S, Saha KB, Jayswar H (2022). Monitoring of the village malaria workers to conduct activities of malaria elimination demonstration project in Mandla. Madhya Pradesh Malar J.

